# Impact of educational games on academic outcomes of students in the Degree in Nursing

**DOI:** 10.1371/journal.pone.0220388

**Published:** 2019-07-29

**Authors:** María-José Castro, María López, María-José Cao, Mercedes Fernández-Castro, Sara García, Manuel Frutos, José-María Jiménez

**Affiliations:** 1 Nursing Faculty, University of Valladolid, Valladolid, Spain; 2 Endocrinology and Clinical Nutrition Research Centre (ECNRC), University of Valladolid, Valladolid, Spain; 3 University Hospital Clinic of Valladolid, Valladolid, Spain; Brigham Young University, UNITED STATES

## Abstract

**Objective:**

The aim of using the game-based tool Kahoot! was to evaluate and reinforce the contents taught in the subject of Management and Administration of Nursing, Ethics and Health Legislation Services included in the Degree in Nursing, during the 2016–2017 academic year.

**Methods:**

A prospective quasi-experimental study was carried out on a sample of 116 students. 10 multiple-choice questions were designed, with only one possible correct answer and a 20-second-limited response time for each of the questions. Four of these questions previously answered in the classroom using this game were chosen (20% of the exam). Each one of them corresponded to one unit of the topics taught in the subject.

In order to participate in the educational game, students needed their smartphones or electronic devices. After completing the game, the students’ satisfaction level derived from its use was assessed.

**Results:**

The correct answer rate in the educational game was greater than 50% for all questions except for one, in which the rate was 28.8% (P<0.05). Response time as related to score presented statistically significant differences, and higher scores for those questions with lower response time (P<0.001).

The questions included in the final test which had been previously answered using Kahoot! showed a significantly higher difficulty index than the rest of the final exam questions (P<0.05). Question 3 was the easiest, while being the one in which the highest-scoring students obtained more wrong answers.

For the students this tool was easy to use (89.6%) and they positively valued the content acquisition and comprehension, as well as the teacher-student interaction (P<0.05).

**Conclusion:**

The implementation of educational games which consider response time and correct answers favors competitiveness and motivates students to actively participate in their learning process.

## Introduction

The use of simulation games for the Degree in Nursing is increasingly common. Its usage aims to promote learning, self-efficacy and confidence in clinical situations, as well as to help students to acquire skills in empathy, critical thinking, patient safety, and clinical practice [1–5].

Students’ learning needs have varied due to the influence of new technologies [6], with games being a feasible option for getting the attention of students while achieving a deep learning of information [7], mainly through lab simulations or virtual serious games [8].

The use of multimedia resources is presented to teachers as an opportunity to gain motivation-based learning [9], fun [10], repetition-based learning, experience and reflection [11]. It could be said that the implementation of games in the classroom favors reflective practice, decision-making, new ways of thinking, acting and role-playing that helps students to understand complex concepts [12,13].

Some studies have detected the need to improve the academic learning environment [14], which is often oriented towards the technical and rational side of the profession more than to the teaching of professional care. This situation is sometimes hindered by an increase in the number of students and by the lack of resources [15]. In order to improve this aspect, it is necessary to involve students in their learning process in an active manner, away from the passive attitude of master classes, and supported by oral and written reflection [16]. Debates, brainstorming, group discussions and open questions represent some of the interactive methods that can be included in the educational practice so as to promote learning [17]. Another interesting measure would be the creation of small work groups, in which a guided learning oriented to specific needs could be developed [18].

Within this framework, the University of Valladolid (Spain) favors this type of methodological strategies, encouraging and supporting the design and development of teaching projects which aim to improve the training and qualification of students. The implementation of new educational methodologies can foster motivation and participation, thus stimulating a way of learning in which students internalize contents and understand them through their own experimentation and observation [19]. Serious games favor socialization as well as teacher-student and student-student interaction. Moreover, teachers have the opportunity to choose if they prefer to conduct group work or individualized work in order to promote cooperation and help among students [10]. In fact, students’ professional future relies on creating a good work team, in which each member contributes his/her originality and creativity within a common objective. In addition to promoting learning, educational games also favor immediate feedback and improve collaboration among students [7].

Within the range of active learning games, it is necessary to consider classroom response systems (CRS) or audience response systems (ARS), also known as clickers. These systems are bidirectional devices which allow the creation of questions and the management of the answers, which are projected on a monitor in the form of a histogram or bar chart. The use of clickers in the classroom promotes student stimulation in the classes through a more successful learning experience as it increases participation and attention [20]. These clickers are expensive tools which require specific software. Moreover, their control command system limits the number of participants in the game. In order to solve these problems, there are web pages—such as Kahoot!—that operate on the internet and allow the use of smartphones, tablets and computers instead of complex controls and software. Kahoot! was developed in 2013 by Professor Alf Inge Wnag [21] and is a free student response system which favors the use of games through questions and multiple-choice answers. In this learning tool, students use their mobile phones or other electronic devices to get connected and answer the questions posed by the teacher. The game can be played individually or by teams, through the projection of questions and answers on the virtual platform. Students will obtain a better score depending on their speed of response and, of course, on the highest number of correct answers [22]. Certain competitiveness is generated by having a limited response time and by the challenge of achieving better scores and results in the game than the rest of the classmates. This can contribute to learning in a positive way [23].

An increase in the number of digital games, as well as their inclusion as teaching materials, makes it necessary to assess them as learning tools. The identification of educational dynamics which facilitate learning—both at a methodological level and at a digital level—will introduce better training programs at the curricular level [24] and will also allow a validation of the learning outcomes [7].

Gallegos et al. point out that studies on educational games in nursing are scarce. Therefore, research on this topic should be conducted in order to detect which educational content is most suitable for these games, analyze whether they promote students’ active participation and motivation, and compare the learning outcomes [21].

This paper presents an assessment of the acquisition of contents using the game-based tool Kahoot!. The contents correspond to the course in Management and Administration of Nursing, Ethics and Health Legislation Services included in the Degree in Nursing. In addition, it is also intended to analyze the students’ satisfaction resulting from the use of this educational method.

## Materials and methods

### Study design

A prospective quasi-experimental study was carried out on a sample of 116 students, 29 men (25.7%) and 87 women (74.2%), with an average age of 21.5 years. They were third-year students enrolled in a course in Management and Administration of Nursing, Ethics and Health Legislation Services, included in the Degree in Nursing at the University of Valladolid (Spain), during the 2016–2017 academic year, in which 120 students were enrolled.

This course is taught during the third year of the degree, in the first semester, and is equivalent to 6 European Credit Transfer and Accumulation System (ECTS) credits. The teaching method is based on master classes, along with the development of classroom practices in which students actively participate. Upon completion of each of the four theoretical units that constituted the contents of the subject, the game-based learning tool Kahoot! was used to reinforce and evaluate them individually with the subsequent resolution of the questions in the class.

The final assessment of this subject was conducted according to the academic calendar of the University of Valladolid, one month after the use of Kahoot! in the classroom. The final exam consisted of 20 multiple-choice questions (70%)—in which our research is based on—and of a practical group project (30%).

### Game-based learning tool

The game-based learning tool Kahoot! was implemented during the 2016–2017 academic year as an innovative teaching method, with the aim of improving already acquired skills and competences and continuous assessment. Considering the contents which had to be evaluated in the course, 10 multiple-choice questions were designed, with only one possible correct answer with a limited response time of 20 seconds.

The 10 questions evaluated with Kahoot! S1 Table. correspond to the theoretical contents taught in the 4 units of the subject and were distributed as follows: 2 questions corresponding to unit 1, 3 questions corresponding to unit 2, 2 questions corresponding to unit 3 and 3 questions corresponding to unit 4.

Before starting to use the educational game, the students were explained its instructions and given the possibility of either downloading the application on their mobile devices or using it online. When each of the teaching units was completed, the students were invited to actively participate in the classroom using Kahoot! in order to encourage interaction, competitiveness, skill and competence acquisition and doubt resolution.

### Variables analyzed

The correct answer rate for each question, the response time (in seconds), and the score obtained in each of the questions were analyzed in order to conduct a continuous assessment of the acquisition of educational contents for each of the teaching units. Score is calculated depending on the speed of response and on the time limit for each question. Therefore, calculation is determined as follows:

- All questions are worth 1000 points if answered in under 0.5 seconds.
- To calculate points, round (1000 * (1 –(([response time] / [question timer]) / 2)))

The final test of the subject consisted of 20 questions related to the 4 thematic units. In this final test, 4 questions (20% of the exam) of those previously answered in Kahoot! were included. The difficulty and discrimination indexes were determined in every question of the final test.

The difficulty index was provided by the Information and Communications Technology Service of the University of Valladolid, and calculated as follows:

- Limit: 30% of the total number of students attending the exam.
- Correct answers (strong): Number of correct answers to the questions among the n best students per score, where n is determined by the value of the calculated ‘limit’.
- Correct answers (weak): Number of correct answers to the questions among the n students with worse score, where n is determined by the value of the calculated ‘limit’.
- Difficulty = [(correct answers (strong) + correct answers (weak))* 100] / 2 * limit

Then, the discrimination index was calculated as follows:

- Discrimination = [2 * (correct answers (strong)—correct answers (weak))] / 2 * limit

An assessment of the satisfaction level from the use of this game-based tool was conducted at the end of the course, prior to the final exam. It was carried out through a survey using Kahoot! and was composed of 10 questions, which assessed its ease of use, interaction between students and teachers, motivation for the course and whether the learning tool facilitated the acquisition of the contents previously studied in class. Depending on the question type, the response options were Yes/No or a four-score scale (1 = dissatisfied, 2 = not really satisfied, 3 = quite satisfied, 4 = very satisfied) to measure satisfaction.

All students were over 18 years of age and participated voluntarily in the study after giving their verbal consent, according to the current legislation, Organic Law 3/2018 of December 5, on Personal Data Protection and digital rights guarantee. Their anonymity was always preserved as no personal or socioeconomic data were collected, as stated in the Teaching Innovation Project approved by the University of Valladolid entitled "INTERDISCIPLINARY GAMIFICATION", with code: PID74.

### Statistical analysis

The collection of data was conducted through the preparation of an anonymized data collection table using Excel (Microsoft Office 2013). Then, data were recorded and exported by a single researcher and analyzed using the statistical program IBM SPSS Statistics 24.0 for Windows.

On the one hand, quantitative variables were presented as average ± standard deviation and their normality was established by the Kolmogorov-Smirnov test. On the other hand, qualitative variables were described using absolute and relative frequencies (percentages).

In order to study the association between qualitative variables, the Chi-square test along with Fisher’s exact test or the likelihood ratio test were used, depending on the conditions for their application. Either the Student’s t-test or the Mann-Whitney U test was used, depending on the conditions for the application, to study the differences between means for 2 groups. For more than 2 groups, moreover, either the ANOVA or the Kruskal-Wallis H test was used. The level of significance for all the tests was P≤ 0.05.

### Results

In each of the 10 questions analyzed, the percentage of correct answers, the response time and the score obtained were assessed.

Table 1 shows the results obtained in each question of the quiz. A correct answer rate greater than 50% was observed in all questions except for question 10, in which the correct answer rate was 28.8% (P<0.05).

**Table 1:** Analysis of content-related responses with Kahoot!.

Questions. n = 116	Correct answers (%)	Response time (seconds)	Score
Question 1	67.2	9.03 ± 5.75	488.56 ± 354.10
Question 2	82.8	7.99 ± 5.75	643.68 ± 317.41
Question 3	55.2	6.31 ± 5.04	452 ± 417.61
Question 4	56.1	7.60 ± 6.01	452.07 ± 410.57
Question 5	62	7.64 ± 5.11	494.10 ± 398.86
Question 6	89.8	4.71 ± 3.61	786.16 ± 279.35
Question 7	44.1	8.62 ± 5.20	330.98 ± 383.14
Question 8	59.3	7.47 ± 5.29	475.89 ± 407
Question 9	66.1	4.90 ± 3.67	568.06 ± 415.07
Question 10	28.8	7.66 ± 5.53	230.30 ± 369.37
Mean	61.14	7.36 ± 5.41	496.71 ± 397.86

Questions 2 and 6 showed the highest correct answer rate—82.8% and 89.8% respectively—, and the highest score compared to the rest of questions—643.68 ± 317.41 and 786.16 ± 279.35—(P<0.05).

When comparing correct answers with respect to response time, only question 4 exhibited a significant correlation (P<0.001). As previously said, question 6 showed the highest correct answer rate (89.8%)—with the shortest response time—and the highest score. However, question 10 showed the lowest correct answer rate (28.8%)—with an average time similar to the rest of questions—and the lowest score.

Response time as related to score in each of the questions analyzed presented statistically significant differences in all of them. In addition, higher scores for those questions with lower response time were observed (P<0.001).

The difficulty index for the final exam questions of the course was calculated. The final exam consisted of 20 questions, the first four of which had been previously answered using the dynamics of the Kahoot! quiz game. Table 2 shows the results of the difficulty index, being the results with a difficulty index closer to 100% those corresponding to the easiest questions to answer. It can be observed in the table that the easiest question of the final exam was question 3, which had been previously answered in class with Kahoot!. The other first three questions—also included in the Kahoot! quiz game played in class—showed a significantly higher difficulty index than the rest of the exam questions (P<0.05).

**Table 2:** Difficulty and discrimination indices for the final exam questions.

Questions (n = 116)	Difficulty Index (%)	Discrimination Index (%)	P-value
Question 1	93.44	0.13	0.256
Question 2	93.44	0.13	0.245
Question 3	98.44	-0.03	<0.05
Question 4	88.54	0.17	0.198
Question 5	64.90	0.45	0.211
Question 6	93.44	0.07	<0.05
Question 7	78.85	0.42	0.231
Question 8	29.06	0.20	0.189
Question 9	28.85	0.32	0.112
Question 10	47.91	0.26	0.223
Question 11	62.91	0.29	0.187
Question 12	86.98	0.26	0.213
Question 13	51.66	0.26	0.261
Question 14	72.75	0.26	0.115
Question 15	43.44	0.30	0.161
Question 16	3.23	0.05	0.118
Question 17	38.54	0.07	0.113
Question 18	46.87	0.22	0.199
Question 19	74.16	0.39	0.235
Question 20	41.98	0.21	0.185
**Mean**	61.96	0.22	

However, question 16—not included in the Kahoot! quiz game played in class—showed the lowest item difficulty index (3.23), determined by a high number of blank answers.

The discrimination index tracks the difference between the proportion of ‘good’ students (high scoring group) who answered correctly to a question and the proportion of ‘poor’ students (low scoring group) who answered correctly to the same question. Thus, high and positive discrimination indices indicate that many more students from the high scoring group answered the question correctly compared to the students from the low scoring group. Question 3 was the easiest question to answer and the only one with a negative discrimination index.

Table 3 shows the results obtained in the four first questions of the final exam of the course separately. As previously mentioned, these questions were included in the Kahoot! quiz game played in class before the exam. It was observed that question 3 had the highest proportion of correct answers in both the final exam and the Kahoot! quiz game played in class. Moreover, the proportion of correct answers to each of the four first questions was higher in the final exam than in the Kahoot! quiz game, being the difference between them not statistically significant.

**Table 3:** Comparison between the correct answers in the Kahoot! quiz game and in the final exam.

Final exam questions	Difficulty Index	Correct answers in the Kahoot! quiz game	Correct answers in the final exam	
		(%)	(%)	P-value
Question 1	93.44	67.2	95.85	0.365
Question 2	93.44	82.8	94.88	0.122
Question 3	98.44	55.2	99.04	0.253
Question 4	88.54	56.1	91.69	0.321

Kahoot! was used to evaluate and enhance the contents taught in the lectures of the subject. A total of 67 students completed the survey (57.75%). All participants considered that Kahoot! was an easy-to-use tool and 89.6% of them would recommend its incorporation in other courses of the degree. The students were asked if they would include the results obtained with Kahoot! as part of the final grade of the subject. 32.3% agreed, while 22.6% preferred the evaluation of content only by means of a final exam.

**Fig 1:**
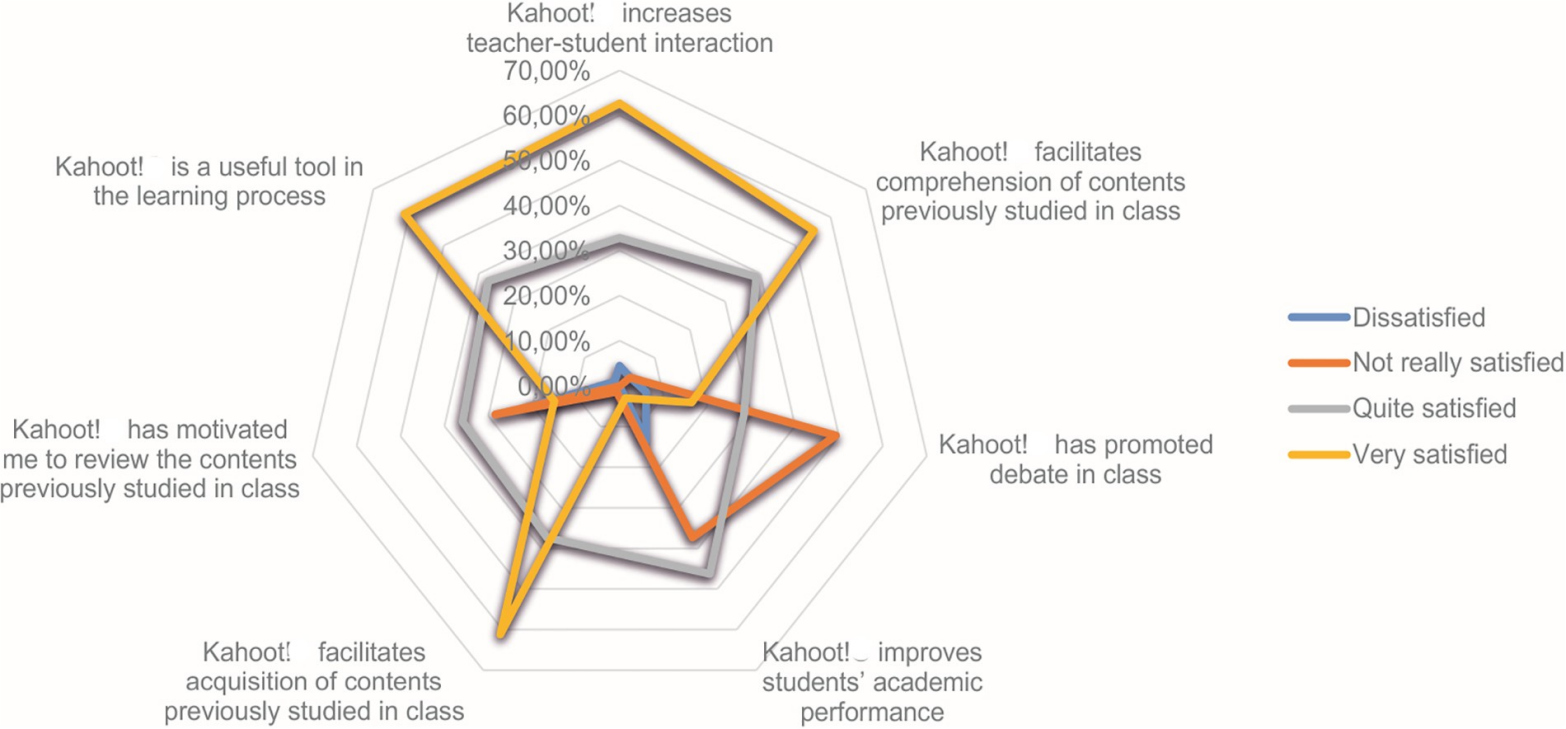
Radar chart representation of the students’ satisfaction level with Kahoot!.

The rest of the items included in the survey provided the students with a four-score scale (1 = dissatisfied; 2 = not really satisfied; 3 = quite satisfied; 4 = very satisfied). (Fig 1) shows the participants’ responses to each item. The items with the highest percentage of positive responses were those related to content comprehension, and teacher-student interaction (P<0.05).

Table 4 includes the mean satisfaction score, obtaining the highest average score in the assessment of ‘Kahoot! is a useful tool in the learning process’, followed by ‘Kahoot! increases teacher-student interaction’.

**Table 4:** Analysis of mean satisfaction score.

Items	Mean satisfaction score
1. Kahoot! increases teacher-student interaction	3.37 ± 0.68
2. Kahoot! facilitates comprehension of contents previously studied in class	3.11 ± 0.69
3. Kahoot! has promoted debate in class	2.66 ± 0.78
4. Kahoot! improves students’ academic performance	2.37 ± 0.79
5. Kahoot! facilitates the comprehension of contents previously studied in class	3.22 ± 0.84
6. Kahoot! has motivated me to review the contents previously studied in class	2.4 ± 1
7. Kahoot! is a useful tool in the learning process	3.44 ± 0.5

## Discussion and conclusions

In this work about the use of the teaching methodology with the Kahoot! tool in a Nursing Degree course, it is worth noting that the students liked Kahoot! since they regarded it as useful and motivating to participate more actively in their learning process.

Only two questions of the quiz—question 2 and question 6—showed the highest correct answer rate, being the latter the one which registered the lowest response time in the quiz. Response time in Kahoot! quiz games is a key element in the Degree in Nursing, because in addition to promoting competitiveness, it is an essential factor for solving problems in the clinical practice of healthcare professionals [25].

One of the main advantages of using Kahoot! in class is that it provides a response time and a ranked score based on the number of correct answers, promoting a certain degree of competitiveness among students.

Overall, the use of clickers in class increases student engagement and participation by promoting content acquisition and comprehension and critical thinking skills. Moreover, as results are shown immediately, students have the opportunity to discuss results and solve doubts forthwith [20]. Clicker-based learning technologies as Kahoot! are beneficial to students because they are easy to use and they provide real-time feedback within a safe environment [26]. Nevertheless, there are students who prefer passive learning and are resistant to in-class games [7].

The results of our study show that the four Kahoot! questions included in the final exam were significantly the easiest questions for students. We consider that incorporating in-class games into the Degree in Nursing might be a useful educational strategy to encourage learning within a low-risk learning environment [27]. This observation is in keeping with that of Strickland et al. These authors reported that nursing students obtained 15 percent more correct answers in their final exams compared to the previous year, when an in-class game had not been implemented as a comprehensive review strategy yet. Moreover, students responded positively to the use of educational games in the learning process [11].

Our findings in this study are consistent with those of Corell et al., who found that learning with competition improved the students’ academic results, and competitive learning tools motivated them to participate more actively in their learning process [23].

One of the Kahoot! questions included in the final exam presented the highest difficulty index (question 3), since it achieved the highest correct answer rate. Interestingly, we found however that the high scoring students in the final exam obtained more wrong than correct answers for that question compared to those in the lowest-scoring group. This observation suggests that students who master the subject content are overconfident about easy questions, and therefore they obtain worse results than those who have not studied the subject in depth.

The results of this study show that it is necessary to think carefully about all elements involved in education before taking a decision about learning; it is essential to make a critical analysis of the results obtained in Nursing Education [6], as well as to obtain objective and subjective data about learning and teaching outcomes in the biosciences [5] by describing in detail the educational interventions implemented—contents, effective pedagogical solutions, learning contexts and settings and assessment tools [28]. More recently, Kalaian reported that students with higher academic performance obtained higher scores and better academic outcomes, had high levels of intrinsic motivation and experienced low anxiety in exams [18]. Overall, there is a significant relation among academic performance, hours of self-study and motivation [29]. Therefore, it is surprising that high scoring students in our study obtained worse results in the easiest question of the final exam.

Moreover, Salvage-Jones et al. found that the practice-learning materials—designed to ensure content comprehension—were considered ‘very useful’ or ‘useful’ by most of nursing students. Nevertheless, students did not perceive an impact on their assessment outcomes [5]. In our study, however, an improvement in the final assessment was observed, especially in the questions previously studied with Kahoot!.

In general, students showed a high level of satisfaction with Kahoot!, being items 1 and 7 the highest rated in the survey. In other words, students considered that Kahoot! increased teacher-student interaction and was a useful tool in the learning process. Our results are consistent with Boctor et al., who reported that students positively assessed the use of educational games and considered that games were useful learning strategies, which could catch their attention and engage them in the learning process [7].

In addition, despite the educational nature of the Kahoot! tool, most of students in the survey believed that this tool was neither a motivating method for reviewing the contents previously studied in class nor an effective tool for boosting debate. It is essential to point out that the teaching method normally used in theory classes encourages debate and participation among students; for this reason, students did not considered Kahoot! extraordinarily motivating. Our findings in this study are similar to those of Gallegos et al., who reported that game scores and rankings were not a motivating element for nursing students, despite the competitive nature of online games. These authors concluded that nursing students might have a high level of intrinsic motivation, so they may not need a gaming component to improve their performance [2].

Besides, the results of this study indicated that game-based methodologies were perceived as useful tools to increase teacher-student interaction. Nevertheless, the role played by teachers is vital: they should understand the game-based learning teaching practices and feel comfortable with them before implementing in-class games. In addition, teachers should be highly engaged with the students’ learning process and be receptive to them [30]. According to Walker et al., collaborative learning favors satisfaction among nursing students, since it enhances both teacher-student and student-student interaction [31].

Overall, based on this study, we believe that educational games are supplementary tools for education, but they cannot replace traditional teaching-learning methods [4]. Nevertheless, educational games in nursing education should be validated, in order to analyze their viability, make them more attractive and capable of combining theory and practice [12]. Students positively assess immediate feedback given by educational games, as they include a personal response system that increases content acquisition and comprehension, as well as motivation in nursing [2].

The main limitation of this study is the lack of randomness of the selected sample. The response rate was high, although the students were not obliged to answer the questions. On the other hand, the planning of the study was determined by both the schedule of classes and the academic calendar. The design of the quasi-experimental study with a single group allows the researcher to manage the exposition, but it does not include a comparison group. Each subject acts as their own control. There is a possibility that there will be Hawthorne effect of generating a response induced by the knowledge of the participants knowing that they are being studied. Students’ higher academic performance in the final test cannot be attributed exclusively to the use of the Kahoot! because other uncontrolled variables come into play: the student's study time, academic performance, affinity with the taught syllabus, previous knowledge, etc.

In conclusion, a game-based learning methodology could be used to foster the active participation of students and to increase competitiveness among them. Moreover, the content-based questions included both in the final exam and in the Kahoot! quiz game played in class showed the highest response index. Finally, the general satisfaction level of students was very positive, as they consider that game-based learning tools not only improve the learning process and facilitate content acquisition and comprehension, but also promote teacher-student interaction.

## Supporting information

S1 Table Questions evaluated on Kahoot!. (DOCX)
