# Supplementary material for: Impact of educational games on academic outcomes of students in the Degree in Nursing
Source: PLoS One. 2019 Jul 29;14(7):e0220388. doi: 10.1371/journal.pone.0220388 (PMC6663014; doi:10.1371/journal.pone.0220388)
Supplement: S1 Table — (DOCX) [file pone.0220388.s001.docx]

**Supporting Information.**

**S1 Table. Questions evaluated on Kahoot!**

Question 1. Who of these authors represent the theory of scientific management?

Question 2. When do organizations for the safety of people's health appear for the first time?

Question 3. Indicate one of the characteristics of the medical device.

Question 4. Accreditation is a verification process of:

Question 5. From the health business point of view, health is:

Question 6. Which authors defended the concept of ergonomics within the industrial efficiency?

Question 7. Who are the main characters of Health Systems?

Question 8. What kind of cost represents savings in health resources?

Question 9. The analysis of structure is a method of evaluation:

Question 10. What regulation gathers Spanish health protection?
